# Synergistic Influences of Stearic Acid Coating and Recycled PET Microfibers on the Enhanced Properties of Composite Materials

**DOI:** 10.3390/ma13061461

**Published:** 2020-03-23

**Authors:** Dang Mao Nguyen, Thi Nhung Vu, Thi Mai Loan Nguyen, Trinh Duy Nguyen, Chi Nhan Ha Thuc, Quoc Bao Bui, Julien Colin, Patrick Perré

**Affiliations:** 1 CentraleSupélec, Laboratoire de Génie des Procédés et Matériaux, Université Paris-Saclay, SFR Condorcet FR CNRS 3417, Centre Européen de Biotechnologie et de Bioéconomie (CEBB), 3 rue des Rouges Terres, 51110 Pomacle, France; julien.colin@centralesupelec.fr; 2 Faculty of Material Science and Technology, University of Science-VNU, Ho Chi Minh city 700000, Vietnam; nhungvt0696@gmail.com; 3 Institute of Research and Development, Duy Tan University, Da Nang 550000, VietNam; thi.mai.loan.nguyen.94@gmail.com; 4 Center of Excellence for Green Energy and Environmental Nanomaterials (CE@GrEEN), Nguyen Tat Thanh University, 300A Nguyen Tat Thanh, District 4, Ho Chi Minh City 755414, Vietnam; ndtrinh@ntt.edu.vn; 5 Sustainable Developments in Civil Engineering Research Group, Faculty of Civil Engineering, Ton Duc Thang University, Ho Chi Minh City 700000, Vietnam; 6 CentraleSupélec, Laboratoire de Génie des Procédés et Matériaux, Université Paris-Saclay, 8-10 rue Joliot-Curie, 91190 Gif-sur-Yvette, France

**Keywords:** pretreatment calcium carbonate, composite artificial marble, stearic acid, recycled PET fibers, fiber-reinforced composite, SEM analysis

## Abstract

This study aims to produce novel composite artificial marble materials by bulk molding compound processes, and improve their thermal and mechanical properties. We employed stearic acid as an efficient surface modifying agent for CaCO_3_ particles, and for the first time, a pretreated, recycled, polyethylene terephthalate (PET) fibers mat is used to reinforce the artificial marble materials. The innovative aspects of the study are the surface treatment of CaCO_3_ particles by stearic acid. Stearic acid forms a monolayer shell, coating the CaCO_3_ particles, which enhances the compatibility between the CaCO_3_ particles and the matrix of the composite. The morphology of the composites, observed by scanning electron microscopy, revealed that the CaCO_3_ phase was homogeneously dispersed in the epoxy matrix under the support of stearic acid. A single layer of a recycled PET fibers mat was pretreated and designed in the core of the composite. As expected, these results indicated that the fibers could enhance flexural properties, and impact strength along with thermal stability for the composites. This combination of a pretreated, recycled, PET fibers mat and epoxy/CaCO_3_-stearic acid could produce novel artificial marble materials for construction applications able to meet environmental requirements.

## 1. Introduction 

Polyethylene terephthalate (PET) is one of the most common plastics widely used in packaging and bottle fields, leading to a massive amount of PET waste to the environment every year [1,2]. The reuses of PET waste are essential, preventing environmental pollution, and limiting the exploit plastics derived from petroleum. From the late twentieth `century to the current, the PET bottles waste has been recycled and widely used as efficient reinforced material nowadays for construction worldwide [3,4,5,6,7].

One of the most important applications of recycled PET fibers is to reinforce building materials to enhance insulation performance, mechanical properties, economical and friendly environment. Notably, it was used as adhesive for polymer concrete, filler for lightweight concrete [8], potential filler for polymer composite and concrete reinforcement material [9,10].

Consequently, recycled PET fibers-reinforced concretes shows an excellent performance in mechanical properties and crack resistance, due to the excellent physical properties of the recycled PET. Additionally, different geometric patterns of waste PET were added to cement-based composite to improve the mechanical bond behavior and control of plastic shrinkage cracking [11], and enhance the long-term durability of cement composites [12]. Currently, no more reports used recycled PET fibers to reinforce the artificial marble materials, which are usually produced from polyester resins acting as a binder, and inorganic fillers such as natural marble powders, or CaCO_3_ particles combined with some additives to create the similar natural marble materials. The manufacturing process of artificial marble often uses molding, compression and heating curing methods [13]. Over the past two decades, there were many studies on composite materials, on thermosetting resins with inorganic fillers to create artificial composite materials. For example: Chen et al. conducted the effect of titanate-based compatibilizer on the physical properties of composite materials based on epoxy resin and CaCO_3_ filler [14]. CaCO_3_ nanoparticles with different amounts and silane coupling agents have been used to significantly improve the mechanical and thermal properties of epoxy-based, composite materials [15], increasing about 13.5% for increasing flexural and about 6.1% for elastic modulus [16]. In the literature, many studies worked on the composite materials, which specialize in the fundamental interfaces in composites. The interfacial aspects are the surface treatment of CaCO_3_ particles by stearic acid. Stearic acid is a surfactant with a hydrophobic tail and a hydrophilic head. It was used to modify the surface of CaCO_3_ particles via a chemical reaction to improve the hydrophobic properties [17,18]. It also effects on the physico-chemistry and mechanical properties, as well as the thermal stability of composites [19,20]. So far, the PET fibers only used to reinforce the concretes, and in fact, no investigations have studied using recycled PET fibers to reinforce within artificial marble materials and deeply analyzed their properties. Therefore, this work is to use the recycled PET fibers mat as a reinforcement phase to produce the artificial marble materials derived from epoxy resin and CaCO_3_ filler modified by stearic acid. The influence of stearic acid and the recycled PET fibers mat on the morphology, mechanical and thermal properties of the artificial marble is investigated. The obtained results indicated that the stearic acid monolayer can effectively coat CaCO_3_ particles, and acts as a coupling agent in the epoxy matrix. The recycled PET fibers mat pretreated with NaOH solution exhibits a significant enhancement in flexural and impact properties, as well as thermal stability for the artificial marble materials.

## 2. Experimental 

### 2.1. Materials 

Epoxy resin (code YD128S) with a viscosity of 11,500–13,500 cps (25 °C) was purchased from Kukdo Chemical Co., Ltd. (Seoul, South Korea). Triethylenetetramine (TETA), a curing agent, was supplied by Tosoh Co., Ltd. (Tokyo, Japan). The recycled Polyethylene terephthalate (PET) fibers mat (code IC67) with a dimension of 125 × 90 × 4 (mm^3^) was supplied from a recycling company in Vietnam. Calcium carbonate (CaCO_3_) particles with diameters about 2–5 µm were purchased from the Surint Omya Co., Ltd. (Hanoi, Vietnam). Stearic acid in the form of flakes with M_w_ = 284.49 (g/mol) was purchased from Unichem Co., Ltd. (Mumbai, India), and NaOH solid was supplied by Xilong Co., Ltd. (Guangdong, China). 

### 2.2. Recycled PET Fibers Pretreatment

Recycled PET fibers in the form of the mat with a dimension of 125 × 90 × 4 (mm^2^) and about 3.3 g, was pretreated by immersing in NaOH solution 50 (% *w*/*v*) for 3 min at a temperature of 80 °C. Then, they were washed with distilled water until the solution after washing achieves pH = 7. Finally, the samples were dried at 50 °C for 12 h.

### 2.3. Modification of CaCO_3_ Surface by Stearic Acid

The CaCO_3_ particles were dried in an oven at 150 °C for 24 h to remove water moisture. Then its surface was modified by stearic acid according to the following process, where firstly, stearic acid was evenly dispersed in ethanol solvent, and this solution was ultra-sounded for 10 min. Then, the CaCO_3_ particles were added slowly to the above solution, and stirred continuously by a mechanical stirrer at 300 rpm for 6 h and 60 °C. The CaCO_3_ particles after treating with stearic acid were filtered and dried in an oven at 50 °C for 8 h to remove the ethanol solvent. Finally, CaCO_3_ was crushed and sifted with 80 mesh size to obtain fine powders for further artificial marble manufacturing.

### 2.4. Preparation of Epoxy/CaCO_3_/recycled PET Fibers Mat

**Figure 1:**
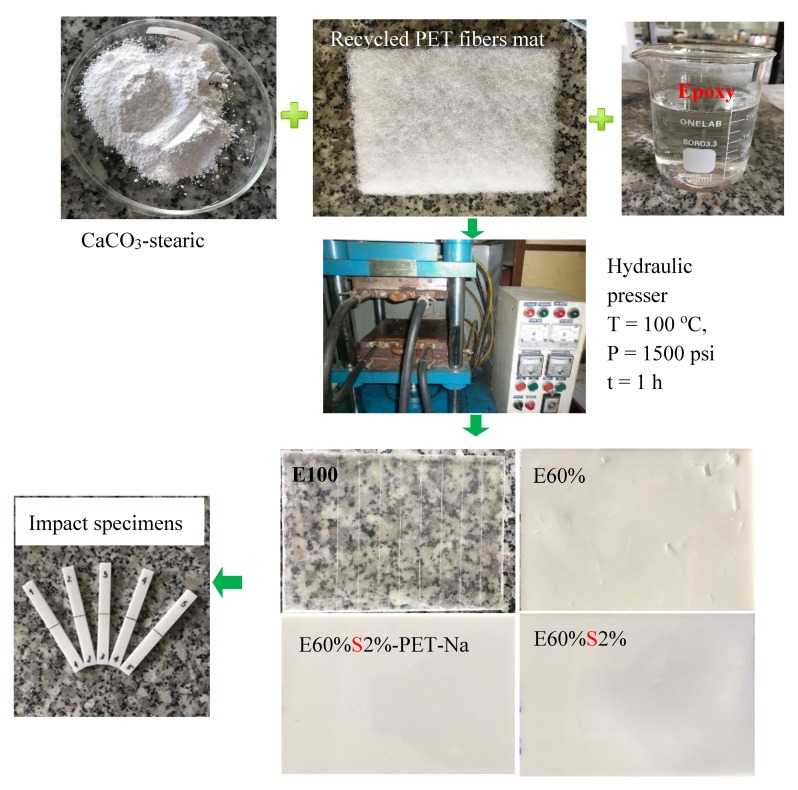
Manufacturing process of cultured marble materials from epoxy/triethylenetetramine (TETA), CaCO_3_ modified by stearic acid and recycled polyethylene terephthalate (PET) fibers mat pretreated with NaOH solution using a heating hydraulic presser.

The artificial marble materials were manufactured using a heating hydraulic presser 16H-R-W (Denmark Hydraulics, Punjab, India). Firstly, the CaCO_3_ particles were slowly dispersed into epoxy resin and stirred with a mechanical stirrer at 300 rpm for 6 h at room temperature. Then, TETA was slowly added into the epoxy/CaCO_3_ mixture, and the speed of the stirrer was increased to 600 rpm for 2 min to the TETA into the epoxy/CaCO_3_ mixture. Then, the mixture was poured into an aluminum mold with a dimension of 120 × 90 × 4 (mm^3^), which contains a layer of recycled PET mat in the middle of the mold. The mold containing the mixture and the recycled PET fibers mat was pressed at a temperature of 100 °C, under a pressure of 1500 psi for 1h. Finally, at the end of the process, the sample was kept stored in the mold at 60 °C for 24 h that allowed us to complete cure and avoid the warping of the artificial marble samples. The manufacturing processing steps were described in Figure 1, and the ingredients of the artificial marble materials were listed in Table 1. 

### 2.5. Material Characterizations

A scanning electron microscope (SEM) was used to assess the dispersion phases of CaCO_3_ in the epoxy resin, and observe the morphology of phases in the artificial marble. The cryo-fractured specimens were prepared using liquid nitrogen and measured by SEM (JSM 6600, JEOL Co., Tokyo, Japan). Fourier Transform-Infrared Spectroscopy (FT-IR) was conducted using a NICOLET 6700 spectrometer (Thermo Scientific Co., Waltham, MA, USA) to analyze the structural changes of materials, especially, to evaluate the interaction between the functional groups of the components in materials in a wavelength range from 400 to 4000 cm^−1^ with a resolution at 4 cm^−1^. Thermogravimetric analysis (TGA) was used to determine the thermal stability of the composite materials with different amounts of components in the sample using a Q500 analyzer (TA Instruments Ltd., New Castle, USA). The TGA measurements were performed in N_2_ nitrogen with a heating rate of 10 °C/min in a temperature range of 25–800 °C, and the mass of each tested specimen was between 19 mg and 21 mg. 

The flexural strength of these composites was performed according to ISO 178: 2010 standard by 3-point flexural measurement using an AG-Xplus Series Precision Universal Tester (Shimadzu Inc., Kyoto, Japan) at a speed rate of 3 mm/min. 

The specimens were measured until completely broken, and the results were the average of at least five specimens of each composite. The Charpy impact strength of the composite was measured according to ISO 179–1 standard, using a Zwick/Roell instrument. The test specimens were prepared with standard dimensions of 80 × 10 × 4 (mm^3^). The impact strength result was calculated based on the average measurement of at least five specimens in each composite.

## 3. Results and Discussion 

### 3.1. Recycled PET Fibers Treatment 

**Figure 2:**
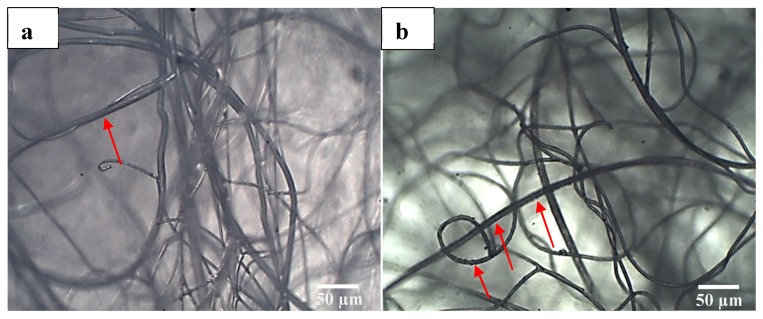
Morphology of recycled PET fibers: (**a**) before and (**b**) after treating with NaOH solution.

SEM images were used to examine the surface morphology of recycled PET fibers before and after treatment with NaOH solution, as shown in Figure 2. The recycled PET fibers became prone to scratches and roughness after being treated with NaOH solution (see red arrows), as compared to the untreated PET fibers, which are a round shape and smooth. This phenomenon was due to the hydrolysis phenomenon in the strong alkaline environment, and the surface of treated recycled PET fibers appears rugged and harsh in having more active functional groups, which improve the interaction between the epoxy resin, as well as other components in the composite.

**Figure 3:**
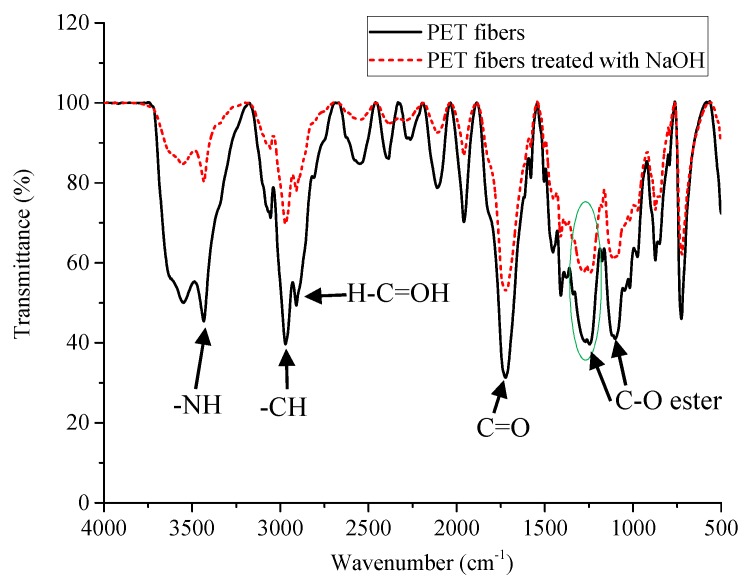
Fourier transform infrared spectroscopy (FT-IR) spectra of the recycled PET fibers before and after being treated with NaOH solution.

FT-IR spectra were also used to verify the changes in the structure of recycled PET fibers after treatment with NaOH solution. In fact, the surface hydrolysis of PET fibers in an alkaline environment (NaOH) did not make a clear change when analyzing with FT-IR spectra as the same observation in a previous study [21]. The vibration peaks of the PET fibers before and after being treated with NaOH were only different a few wavenumbers (cm^−1^). As indicated in Figure 3, recycled PET fibers have two characteristic peaks, at 3547.63 cm^−1^, corresponding to –OH stretching vibration, and at 3434.04 cm^−1^, corresponding to the variation of –NH stretching in polyamide, which was blended at a small part with recycled PET to shape and improve some properties for recycled PET fibers [22,23]. Two other peaks at 2960 and 2900 cm^−1^ correspond to CH_3_ stretching and CH_2_ stretching vibrations, respectively. The most apparent change was observed that the peak of the recycled PET fibers at 1245.91 cm^−1^ was split into two peaks at the 1276.37 cm^−1^ and 1243.66 cm^−1^ wavenumbers. This was due to the hydrolysis phenomenon that cut the PET fibers chains and formed the new –COO groups on the PET surfaces, causing the energy from –C=O to be shared by the adjacent –C–O groups (only a tiny fraction on the surface of the PET fibers). This makes two peaks appear more evident than before surface treatment [24]. 

### 3.2. CaCO_3_ Modification by Stearic Acid

**Figure 4:**
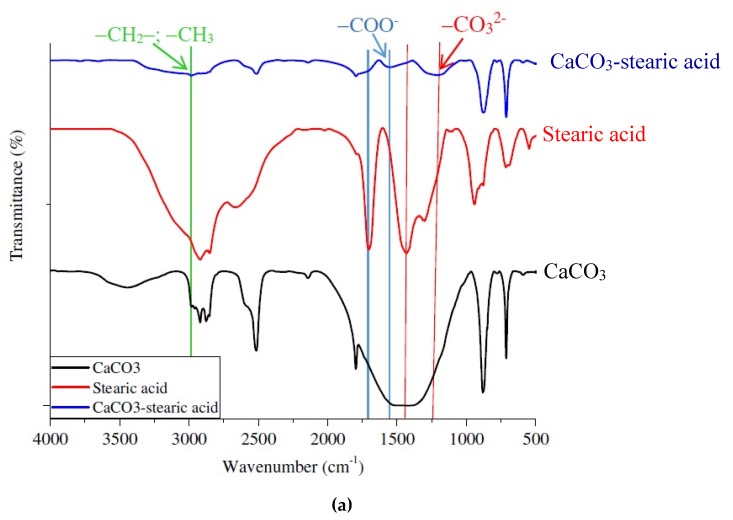

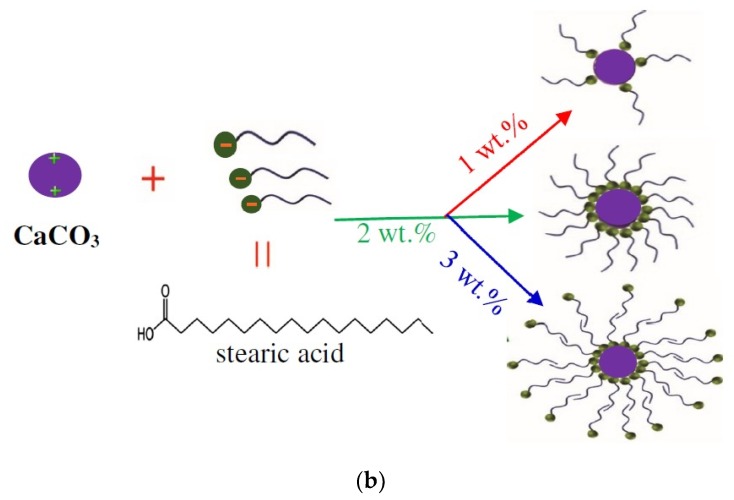
(**a**) FT-IR spectra of CaCO_3_, stearic acid and CaCO_3_ modified by stearic acid, and (**b**) a schema illustration of reaction between CaCO_3_ and stearic acid at different contents.

FT-IR spectra (Figure 4a) were used to evaluate structural changes on the surfaces of CaCO_3_ due to a stearic acid agent. Generally, CaCO_3_ particles have a wide peak at 3449.09 cm^−1^, corresponding to the stretching vibration of –OH groups on the surface of CaCO_3_ [19,25]. Three strong intensity peaks are 1410.64 cm^−1^ of the asymmetric stretch vibration, 883.40 cm^−1^ of the out-of-plane bending absorption vibration, and 712.47 cm^−1^ of the in-plane bending vibration for –CO3^2−^ [26]. 

In the case of stearic acid, there was a peak at 2919.25 cm^−1^ corresponding to the extension vibration of the –C–C– chain. The peaks from 2873,03 cm^−1^ to 2663.57 cm^−1^ were characteristic vibrations of –C–H [27], and a peak at 1702.99 cm^−1^ corresponds to –C=O vibration in the –COOH group. The FT-IR spectrum of CaCO_3_ modified by stearic acid keeps the two characteristic vibrations of –CO_3_^2−^ at 875.12 cm^−1^ and 713.05 cm^−1^, but the peak at 1410.64 cm^−1^ was shifted to 1222.96 cm^-1^. A peak at 1702.99 cm^−1^, the typical peak of the –COOH group, was shifted to 1551.89 cm^−1^, because it lost H to convert into –COO– [28]. Moreover, there was a new peak at 2985.50 cm^−1^ representing for –CH_2_– and –CH_3_ vibration, corresponding to the carbon chain of stearic acid. The main mechanism of the modification process was illustrated in Figure 4b that the stearic acid was adsorbed on the surface of CaCO_3_ particles at different contents via a chemical reaction between the –COOH groups and calcium cations. At the end of the process, the CaCO_3_ particles were coated by a monolayer of the hydrophobic fatty oil chains of stearic acid to form as a core-shell structure [29]. However, the core-shell structure and properties were mainly dependent upon the amount of stearic acid used to cover enough CaCO_3_ particles [30]. Additionally, the optimization of the stearic acid amounts on the properties of artificial marble materials will be discussed in Section 3.4.2. 

### 3.3. Morphology of Artificial Marble Materials 

**Figure 5:**
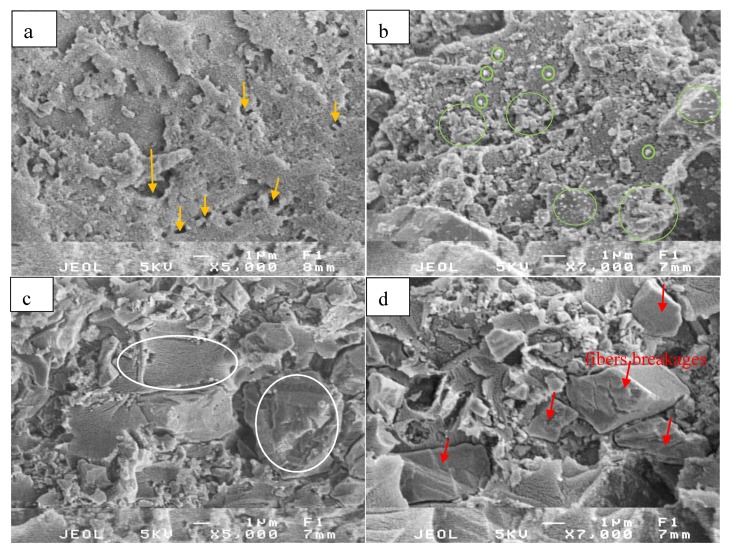
Ruptured surface morphology of (**a**) epoxy resin, (**b**) E60%, (**c**) E60%S2%, and (**d**) E60%S2%-PET-Na.

The SEM images (Figure 5) allow an evaluation of the fractured surface morphology and dispersion of the CaCO_3_ phase in epoxy resin. It was useful to evaluate effective stearic acid agent on the morphology of artificial marble materials. Figure 5a was an SEM image of epoxy/TETA (9:1 wt %), showing a single phase, but the surface was not homogeneous, and contained more large pores (see yellow arrows), which could form during the curing process between epoxy and TETA. The SEM morphology of the ruptured surface of the sample containing unmodified CaCO_3_ particles (E60%) was shown in Figure 5b. The interface between the epoxy matrix and unmodified CaCO_3_ particles was observed and exhibited a poor adhesion between the CaCO_3_ particles and the epoxy matrix, which was proven by the existence of CaCO_3_ particles clusters on the matrix surface. This behavior was formed via the strong interaction between particle–particle of untreated CaCO_3_ particles [31] and they have agglomerated to form the larger particle clusters (see green cycles). The same observation was found in the previous studies of unmodified CaCO_3_ in polybutylene terephthalate [32], polypropylene and HDPE [30]. 

Meanwhile, the morphology of the composite sample with the CaCO_3_ particles modified by stearic acid exhibited a better dispersion of modified CaCO_3_ particles on the epoxy matrix. It was proven by smooth surfaces, as indicated in Figure 5c (see white circles). 

Indeed, the particle–particle interaction disappeared, and the interface between CaCO_3_ particles and epoxy matrix and the agglomerated phenomenon of CaCO_3_ particles were not observed, as indicated in Figure 5b. There was a significant reduction of large voids on the fracture surface. It can also be concluded that the use of stearic acid promotes adhesion and improves the compatibility between the CaCO_3_ particles and the epoxy matrix. This result was due to a monolayer stearic acid, which coated CaCO_3_ particles and acted as coupling agents between CaCO_3_ particles and molecular chains of the epoxy matrix. 

Figure 5d showed the ruptured surface morphology of composite reinforced with recycled PET fibers pre-treated with NaOH solution. Obviously, the surface of this sample was also smooth, and the accumulation behavior of CaCO_3_ particles was not observed, due to the effects of a monolayer of stearic acid. Indeed, CaCO_3_ particles were kept fixing inside the epoxy networks via multidimensional curing under the support of this monolayer stearic acid. Additionally, under NaOH solution, the PET fibers were hydrolysis to form the –COO^−^ functional groups, which interacted with the amine functional groups in TETA and –OH groups of epoxy resin that helps the PET fibers well interacting into this composite.

Interestingly, the PET fibers breakages were observed with different diameters on the fractured surface of composite (see red arrows). The ruptured surface exhibited less voids, and the PET fibers were held tightly within the matrix and no pullout behavior when broken. It shows a good adhesion between CaCO_3_ particles and epoxy matrix that improve the interactions between the epoxy matrix and PET fibers within the composites. At this time, the PET mat was fixed in the center of the artificial marble sheet, which consequently improves the mechanical properties (impact and flexural properties) of this composite.

### 3.4. Mechanical Properties of Artificial Marble Materials

#### 3.4.1. Influent of Stearic Acid and Recycled PET Fibers 

**Figure 6:**
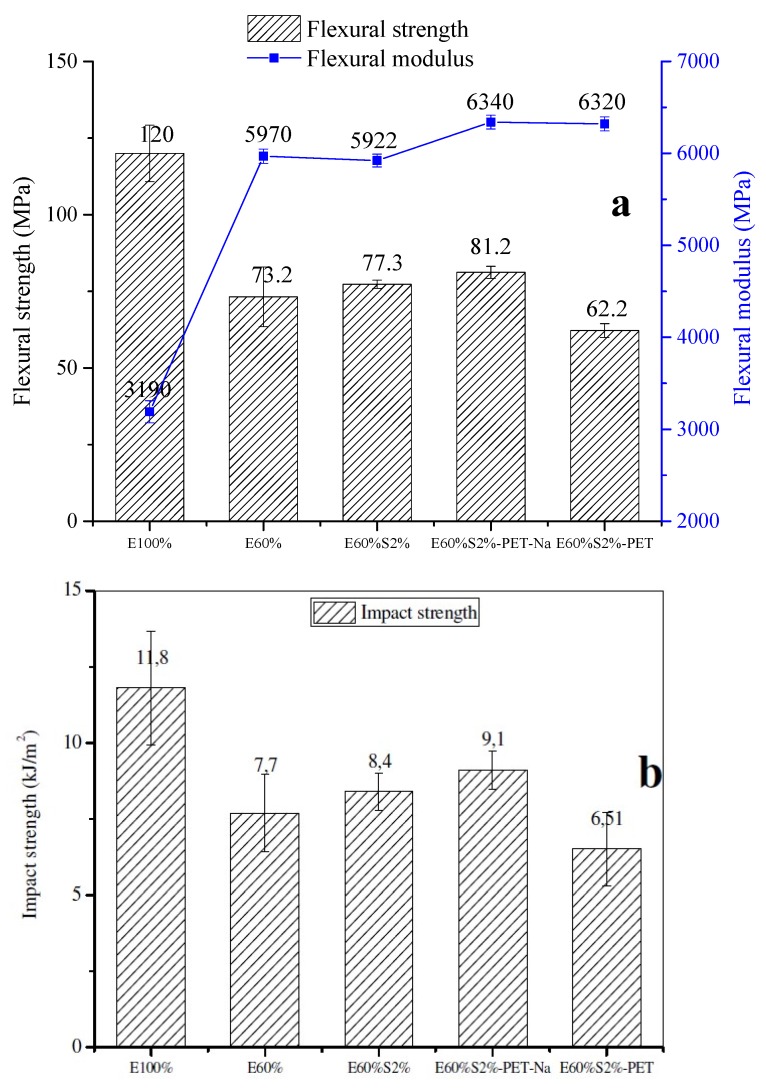
The influence of stearic acid and PET fibers on flexural properties (**a**) and impact strength (**b**) of artificial marble materials.

The flexural properties of artificial marble materials with different ingredients were illustrated in Figure 6a. In the first observation, the flexural property of the epoxy/TETA sample was about 120 Mpa, and 3190 MPa for the flexural modulus. It was decreased to 73 MPa for flexural strength, but significantly increased to 5969 MPa for the flexural modulus of the sample containing 40 wt % CaCO_3_ (E60%). 

Not surprising, the presence of CaCO_3_ leads to hinder the curing process of epoxy and TETA. Acting as an inorganic filler, CaCO_3_ could not disperse evenly in the polymer matrix, leading to accumulation and clustering in the epoxy resin matrix (see Figure 5b), which causes weakness in the interface between the epoxy resin and fillers phase. However, a significant increase of the flexural modulus of this sample came from the stiffness of inorganic CaCO_3_ particles, which was the high mechanical parameter of rigid inorganic fillers compared to polymers. 

The effects of stearic acid agents and recycled PET fibers on the flexural properties of artificial marble materials were also investigated and discussed. Indeed, the use of stearic acid to modify the CaCO_3_ particles’ surface leads to improve the flexural strength of the composite. Especially, in this study, at 2 wt % of stearic acid (E60%S2%) makes a slight increase the flexural strength to 77.4 Mpa, and keeps constant the flexural modulus compared to the sample without using stearic acid (E60%). This result was explained that the stearic acid monolayer acts as the shell, covering the CaCO_3_ particles and reducing the surface energy of these CaCO_3_ particles that helps uniform dispersion CaCO_3_ particles into the epoxy matrix through its bipolar characteristics, as described in Figure 4b. The recycled PET fibers before (E60%S2%-PET) and after being pretreated with NaOH were used to produce the artificial marble composites. In fact, recycled PET-reinforced composite was not treated with NaOH solution, exhibiting the weakness flexural properties through a significant reduction in flexural strength and flexural modulus values about 62 MPa and 6320 MPa, respectively in comparison to the sample without recycled PET fibers’ reinforcement (E60%S2%). Meanwhile, these values of the sample reinforced with recycled PET mat treated with NaOH (E60%S2%-PET-Na) reached the highest values of about 81 MPa for flexural strength and 6430 MPa for flexural modulus, respectively compared to the rest of the artificial marble samples. This was due to the hydrolysis of recycled PET fibers in NaOH solution to form new functional groups (–COO^−^), and the fiber surfaces became rough and rugged, which helped the PET fibers interacting well with epoxy resin, and acted as reinforced agents for this sample. In this case, the PET fibers mat played as an inner backbone to improve the mechanical properties for this composite. Moreover, the PET fibers randomly distributed in composite could prevent the sudden failure, in which the flexural load transferred through the fibers before rupturing the sample. This phenomenon was confirmed with different types of plastics fibers as polyolefin, polypropylene and plastic bag waste [33,34,35,36]. The impact strength of artificial marble samples was plotted in Figure 6b. The epoxy/TETA matrix has an average impact strength of 11.8 kJ/m^2^, while the impact strength of composite containing about 40 wt % of CaCO_3_ content (E60%) was found to decrease remarkably about 1.5 times (average value of 7.7 kJ/m^2^) with the addition of CaCO_3_ into the epoxy/TETA matrix. This phenomenon generally occurs in composites based on the addition of an inorganic filler into a matrix because of the diluting effect of the dispersed phase [37]. The impact strength of the composite containing 2% stearic acid was higher than that of the composites without it (E60%). This result proves that stearic acid acts as a soft agent and improves the dispersion support for CaCO_3_ particles in the matrix. 

In particular, the impact strength of the composite reinforced with recycled PET fiber pretreated with NaOH increased and reached a maximum value of 9.14 kJ/m^2^ when compared to E60% (7.7 kJ/m^2^). This was also significantly higher compared to the composite containing untreated recycled PET fibers (6.5 kJ/m^2^). The results were suitable for the previous studies, which reported the use of fibers in different types of building materials to improve toughness and energy absorption capacity [38,39,40,41,42,43].

**Figure 7:**
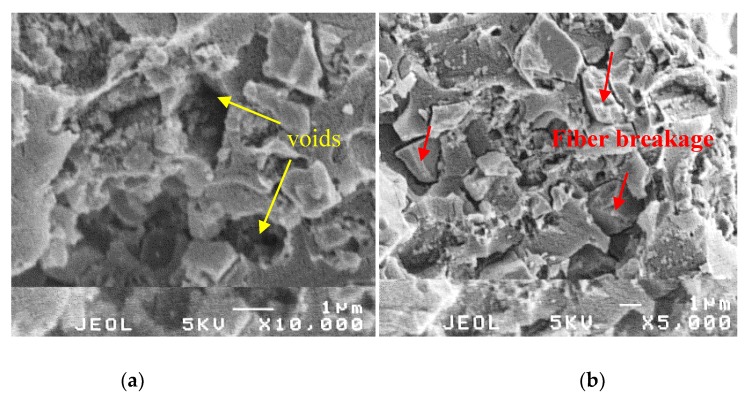
Field emission scanning electron microscopy (FE-SEM) images of (**a**) E60% and (**b**) E60%S2%-PET-Na.

Additionally, the other way to prove the possible reasons which lead to an improvement of the impact strength of the composites, the morphology of fractured surfaces of composites was evaluated in Figure 7. Firstly, it was observed that the aggregation, clumping of CaCO_3_ particles in the presence of a significant amount of CaCO_3_ accumulations (see Figure 5b) and large voids on the surface of the matrix (see Figure 7a).

They are these main contributions that greatly affect the decrease in the impact strength of this composite sample. Meanwhile, a relatively smooth fracture surface was observed in the case of the composites after 2% stearic acid and recycled PET pretreated with NaOH were added into the E60%, suggesting the miscibility and compatibility of Epoxy/TETA matrix and CaCO_3_ particles (see Figure 7b). At this state, CaCO_3_ particles coated by stearic acid, effectively filled the voids, and was uniformly distributed in the epoxy matrix, resulting in better stress transferring across the interfaces in the composites, leading to the improvement of the impact strength of the materials. Therefore, the use of stearic acid and recycled PET fibers treated with NaOH is a current potential way to improve the impact strength of the artificial marble materials. 

#### 3.4.2. Influent of Stearic Acid Contents

**Figure 8:**
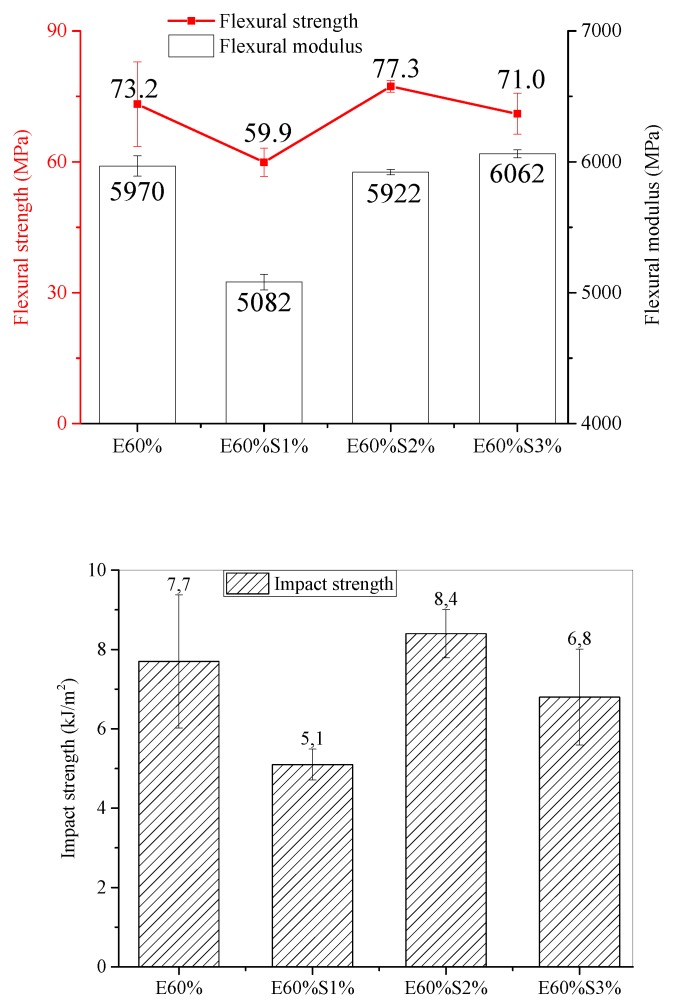
The influence of stearic acid contents on flexural and impact properties of composite materials.

The effective modification of stearic acid on the flexural and impact properties of the composite samples was showed in Figure 8. Both of flexural and impact properties of the sample containing at a low content stearic acid (1 wt %) were lower than the sample without using it (E60%). In this case, the stearic acid has not sufficiently shown its role of thoroughly wetting the surface of CaCO_3_ particles, leading to uneven modification, which causes CaCO_3_ accumulation and clumping as non-modified CaCO_3_ particles part. When the stearic acid content was increased by 2 wt %, it could be seen that the impact strength and flexural properties increased significantly to 8.4 kJ/m^2^ for impact strength, about 77.3 MPa for flexural strength and 5922 MPa for flexural modulus, respectively, compared to those of composite with 1 wt %. This result was due to the fact that stearic acid acted as a surface coating for CaCO_3_ particles, forming a core-shell structure between stearic acid and CaCO_3_ particles, as described in Figure 4b. This reduces the surface energy of CaCO_3_ particles, and helps the uniform dispersion CaCO_3_ particles into the epoxy matrix through this core-shell structure. Also, the significant reduction of the micropores and the interfaces inside the composite also improved the mechanical properties of this material. However, at a high content of stearic acid (3 wt %), its excess amount in the composite created stereoscopic obstacles that hinder the curing of epoxy chains, resulting in flexural strength and impact strength reductions. Particularly, at 3 wt % of stearic acid, while the flexural modulus of this sample was constant, the impact and flexural strength of composites were reduced to 6.8 kJ/m^2^ and 70.9 kJ/m^2^, respectively. Indeed, the residual content of stearic acid in the composite effects the curing process of the epoxy resin, which leads to reducing the mechanical properties of the composite.

### 3.5. Thermal Decomposition of Artificial Marble Composites 

**Figure 9:**
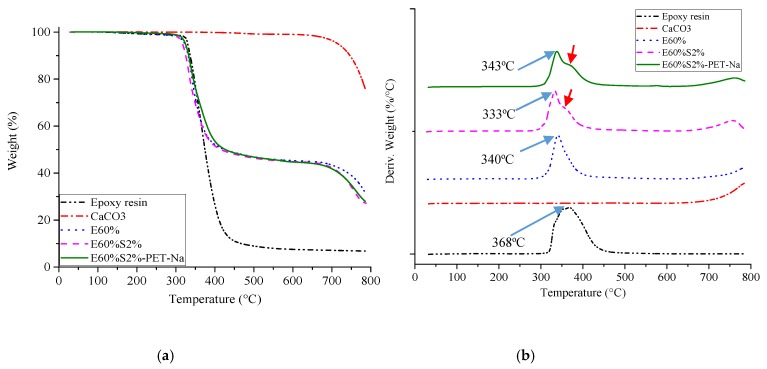
Thermal stability of samples: the TGA curves (**a**) and DTG curves (**b**) of cultured marble materials.

The thermal stability curves of epoxy resin, CaCO_3_ particles, and the composites were shown in Figure 9a. Epoxy resin began to thermally decompose at 320 °C, corresponding to a mass loss of about 93.2% and only a single thermal decomposition step. The CaCO_3_ particles have a very high thermal decomposition temperature, starting at 700 °C, because the test temperature was set up to 800 °C, so it has not shown all the processes of thermal decomposition and mass loss of CaCO_3_ particles [18]. In general, the thermal stability curves of E60%S2%-PET-Na and E60%S2% have been similar to sample E60%. Indeed, two well-defined steps were observed in the TGA curves. The first step was about 320 °C ending at 390 °C, corresponding to the thermal decomposition of epoxy resin with about 55% of mass loss. The second step starts at 695 °C, corresponding to the thermal decomposition of CaCO_3_ particles in samples. At this step, the signal overlapping precludes the direct determination of when the temperature begins to decompose the epoxy resin in each sample. Therefore, the detailed thermal decomposition at the first step of each sample is listed in Table 2. Not surprisingly, the thermal composition of E60% was lower than epoxy; this was due to the poor compatibility between epoxy and CaCO_3_ particles. For the sample E60%S2%, the mass loss occurs at a temperature of 15 °C lower than the epoxy. This was explained by the thermal decomposition of stearic acid in this sample [44]. 

However, when recycled PET fibers pretreated by NaOH were added to this composite, the thermal stability was enhanced to the same temperature of the epoxy matrix. Thus, the reinforcement of recycled PET fibers pretreated by NaOH into composite material has significantly improved the thermal decomposition compared to E60%.

The DTG curves (Figure 9b) showed the most significant mass loss of epoxy at about 368 °C, while the sample E60% has the most significant mass loss at about 340 °C. This result was due to poor compatibility of the E60% sample, resulting in this sample being susceptible to thermal decomposition. Also, the exothermic peak of sample E60%2% was split into 2 peaks; a peak was shifted to a lower temperature at 330 °C, attributing to the physically adsorbed molecules of stearic acid and the surface precipitated calcium stearate in the material. This was reported with an exothermic peak at T > 310 °C in the previous study [18]. The second exothermic peak was attributed to the thermal decomposition of the epoxy matrix. A similar observation was found for the sample 60%S2%-PET-Na, a first peak attributed at 343 °C due to the thermal stability of stearic acid. However, the presence of recycled PET fibers pretreated with NaOH promotes to improve the thermal stability of this sample, which increased by 10 °C compared to E60%S2%, and by 2 °C compared to E60% (without stearic acid). Indeed, the randomly distributed PET fibers in composite could prevent the thermal decomposition of the sample, in which the heat load transferred through the fibers before the thermal decomposition of the sample.

## 4. Conclusions 

This research has successfully produced artificial marble materials based on epoxy resin with a dispersion phase of CaCO_3_ particles modified by stearic acid and reinforced by recycled PET fibers pretreated with NaOH by a bulk molding compound process. Based on the results obtained, some conclusions are drawn as follows: 

The 40 wt % of CaCO_3_ content was used to produce artificial marble materials, 2 wt % stearic acid was the optimal content to modify the surface of CaCO_3_ particles. The reinforcement of the recycled PET fibers pretreated with NaOH led to improved mechanical and thermal stability properties of artificial marble materials.

The changes in composite structure were assessed through the SEM and FT-IR methods. Indeed, stearic acid has created a monolayer, as a shell coating CaCO_3_ particles that improves the dispersion of CaCO_3_ particles in the epoxy/TETA matrix, thereby improving the mechanical properties of composite samples. The recycled PET fibers after being treated with NaOH solution have a good interaction with the matrix, so it showed the role of the reinforcement phase, significantly improving the mechanical properties of the composites.

The surface treatment of CaCO_3_ particles with stearic acid slightly reduces the thermal stability of the composite, however. However, when this sample was reinforced by recycled PET fibers pre-treated with NaOH, its thermal stability was improved. 

## Figures and Tables

**Table 1:** The amounts of different components in the composites.

Materials	Epoxy/TETA (9:1) (g)	CaCO_3_ (g)	Stearic Acid (g)	Recycled PET Fibers Treated by NaOH (g)
E100%	50.0	0.0	-	-
E60%	30.0	20.0	-	-
E60%S2%	30.0	19.6	0.4	
E60%S2%-PET-Na	30.0	19.6	0.4	3.3

**Table 2:** The first step thermal decomposition parameters of composite materials.

Material	Begin Temperature (°C)	End Temperature (°C)	Mass Loss (%)
Epoxy	330	416	93.2
CaCO_3_	695	-	-
E60%	324	381	54.5
E60%S2%	316	364	55
E60%S2%-PET-Na	329	395	54
